# Development and validation of a perioperative risk prediction model for pressure ulcers in neurosurgical procedures: a machine learning approach with protocol compliance metrics

**DOI:** 10.3389/fmed.2025.1600481

**Published:** 2025-07-02

**Authors:** Yaping Wang, Weiguang Yu, Hui Zhi, Kun Shang, Hongmei Yin, Dandan Shan, Xiao Li, Wenxia Li, Xiuru Zhang, Baoli Zhang

**Affiliations:** ^1^Department of Anesthesia and Perioperative Medicine, Henan Provincial People’s Hospital, Zhengzhou, China; ^2^Department of Emergency Surgery and Orthopaedics, The First Affiliated Hospital, Sun Yat-sen University, Guangzhou, China; ^3^Department of Spinal Cord and Spinal Surgery, Henan Provincial People’s Hospital, Zhengzhou, China; ^4^Department of Hypertension and Vascular Disease, The First Affiliated Hospital, Sun Yat-Sen University, NHC Key Laboratory of Assisted Circulation, Sun Yat-Sen University, National-Guangdong Joint Engineering Laboratory for Diagnosis and Treatment of Vascular Diseases, Guangzhou, China

**Keywords:** neurosurgical procedure, nomogram, pressure injury, predictive model, retrospective analysis

## Abstract

**Background:**

This study aimed to develop and validate a nomogram for predicting pressure ulcer (PU) incidence in neurosurgical patients to enhance postoperative risk management.

**Methods:**

A retrospective analysis of 1,020 patients across four tertiary centers (2005–2025) evaluated 20 variables. Propensity score matching (PSM) addressed confounding, while LASSO regression and machine learning identified predictors. Model performance was assessed via AUC-ROC, C-index, and decision curve analysis.

**Results:**

Eight independent predictors of PU were identified: diabetes duration, BMI, albumin, prealbumin, age, hemoglobin, temperature difference, and urinary incontinence. The training set achieved an AUC-ROC of 0.825 (95% CI: 0.797–0.853) with 77% sensitivity and 92% specificity, while the validation set showed an AUC-ROC of 0.800 (95% CI: 0.753–0.847) with 76% sensitivity and 92% specificity. The nomogram demonstrated recalibrated C-indices of 0.833 (training) and 0.826 (validation). Decision curve analysis confirmed significant net benefit across clinical thresholds.

**Conclusion:**

This validated nomogram enables early PU risk stratification, facilitating personalized postoperative interventions. Given its high sensitivity and specificity, the model can be integrated into clinical practice to assist in early identification of high-risk patients, thereby improving patient outcomes through timely interventions.

## Background

Pressure ulcers (PUs) are critical complications in neurosurgical patients, with incidence rates ranging from 8.3 to 23.6% in prolonged procedures (1, 2). Unique risk profiles arise from prolonged immobilization, hemodynamic instability, and intraoperative positioning challenges (3–5). Existing tools like the Braden Scale exhibit limited specificity (52–68%) in surgical settings due to unaddressed confounding biases (6–8), particularly in neurosurgery where selection biases in high-risk cohorts distort risk associations (7, 9, 10).

Recent methodological advancements in causal inference, particularly propensity score matching (PSM), robust confounding adjustment by creating balanced cohorts through counterfactual frameworks (11, 12). By creating balanced cohorts through counterfactual framework estimation, PSM enables quasi-experimental conditions for evaluating treatment-outcome relationships in retrospective data (11, 13). This approach has demonstrated particular utility in surgical outcomes research, with recent studies reporting 25–40% reductions in selection bias when comparing matched cohorts (14, 15). This study pioneers the integration of PSM with machine learning algorithms to develop a neurosurgery-specific PU prediction nomogram. Our methodology: 1. Applies PSM with 1:2 nearest-neighbor matching on 15 covariates to balance PU/non-PU groups; 2. Incorporates protocol compliance metrics as stabilizing weights; 3. Utilizes machine learning-enhanced variable selection to address residual confounding. This hybrid approach addresses three critical gaps in perioperative risk stratification: 1. Mitigation of indication bias in surgical PU attribution; 2. Enhanced generalizability through dynamic intraoperative parameter integration; 3. Translational applicability via protocol adherence quantification.

The resulting model demonstrates superior predictive performance compared to traditional approaches (ΔAUC +0.18), establishing a new paradigm for risk-adjusted outcome analysis in neurosurgical quality improvement initiatives.

## Methods

### Study population

A multicenter retrospective cohort included 1,020 adults (≥18 years) undergoing elective craniotomy at four tertiary centers (2005–2025). Each participating center followed a standardized protocol for pressure ulcer prevention, including scheduled repositioning of patients, the use of pressure-relieving mattresses, and early postoperative mobilization. Adherence to these protocols was quantitatively assessed using a protocol compliance index. Minor variations may exist due to institutional practices, which have been discussed further in the limitations section. The model development followed a structured five-step framework (13), fully adhering to the TRIPOD guidelines (Transparent Reporting of a multivariable prediction model for Individual Prognosis Or Diagnosis) for prediction model development and validation (16).

Patients were excluded if they met any of the following criteria: 1. pre-existing PU or skin breakdown at baseline; 2. emergency craniotomy due to life-threatening conditions (e.g., intracranial hemorrhage, severe traumatic brain injury); 3. intraoperative complications including operative duration exceeding 6 h or blood loss greater than 500 mL (17); 4. postoperative complications such as sepsis (defined as fever >38.5°C for >24 h with positive blood culture), coagulopathy requiring anticoagulation, or unconsciousness/decreased mobility preventing repositioning; 5. comorbidities including end-stage malignancy, advanced heart failure, renal failure, immunosuppressive disorders (e.g., HIV infection, chronic corticosteroid use), or severe malnutrition (albumin <18 g/L or BMI < 16); 6. inability to complete the 7-day postoperative follow-up period (e.g., death within 24 h, transfer to another institution, or loss to follow-up exceeding 10%); 7. concurrent dermatological conditions (e.g., eczema, psoriasis) that could interfere with ulcer assessment; 8. failure to adhere to standardized pressure ulcer prevention protocols (e.g., no alternating positioning schedule or pressure-relief device utilization) or early initiation of advanced wound therapies (e.g., negative pressure wound therapy within 48 h postoperatively); or 9. incomplete medical records (missing >20% key variables) or non-neurosurgical interventions.

These exclusions aimed to minimize confounding variables and focus on analyzing *de novo* PU development in neurosurgical patients with stable perioperative conditions. To address confounding by indication, a two-stage analytical framework was implemented-PSM balanced baseline characteristics between PU and non-PU groups, followed by machine learning model development on the matched cohort. The model development followed a structured five-step framework (13, 16), adapted from TRIPOD guidelines for prediction models (16).

### Covariate selection and matching protocol

A directed acyclic graph identified 20 confounders. These included demographic factors (eg, age, sex, BMI), comorbidity burden, surgical complexity (procedure type, emergency status), preoperative status (serum albumin, BUN, creatinine, baseline Braden Scale score), and institutional factors (center surgical volume, protocol compliance index). Prealbumin (transthyretin), a rapid-turnover nutritional marker, was measured preoperatively to assess acute protein depletion impacting tissue resilience. PSM achieved balance across demographics, comorbidities, and institutional factors. Protocol compliance scores were integrated as stabilizing weights. A Least Absolute Shrinkage and Selection Operator (LASSO) regression selected non-redundant predictors. The optimal *λ* (λ = 0.021) was selected via 10-fold cross-validation using the 1-standard-error rule, prioritizing parsimony while maintaining predictive accuracy. LASSO identified variables (e.g., diabetes duration) with non-zero coefficients.

### Model training and validation

A multivariable logistic regression model was trained on the LASSO-selected predictors. To capture non-linear relationships, an XGBoost model (learning rate = 0.01, max depth = 4) and a neural network (2 hidden layers, L2 regularization) were implemented (18–20). Bootstrapping (1,000 iterations) corrected for optimism bias, and temporal validation ensured stability across time windows (21, 22).

### Statistical analysis

PSM was performed using the nearest neighbor algorithm with a caliper width of 0.2 standard deviations of the logit propensity score. Covariate balance was assessed through SMD, with an absolute SMD < 0.1 considered indicative of adequate balance. The balance assessment was visualized using a Love plot generated with the cobalt package, displaying clinically relevant covariates before and after matching. LASSO regression was used for the initial screening of variables, implemented via the glmnet package (version ≥4.1) in R (version 4.4.3). Variables selected by LASSO underwent backward stepwise regression (retention threshold: *p* < 0.05) to refine clinical interpretability, adjusting for age, sex, and comorbidities. This step excluded two variables (cardiovascular disease, hypertension) that lacked statistical significance (*p* ≥ 0.05) without compromising model performance (ΔAUC<0.01 in sensitivity analysis). Area under the ROC curve (AUC-ROC) and concordance index (C-index) quantified model accuracy. Brier scores and Hosmer-Lemeshow tests assessed agreement between predicted and observed risks. Decision curve analysis (DCA) evaluated net benefit across threshold probabilities (10–90%). Sensitivity analyses included temporal validation through sliding window comparisons and subgroup assessments to verify consistency. All analyses were implemented in R version 4.4.3.

## Results

### Patient characteristics

**Figure 1:**
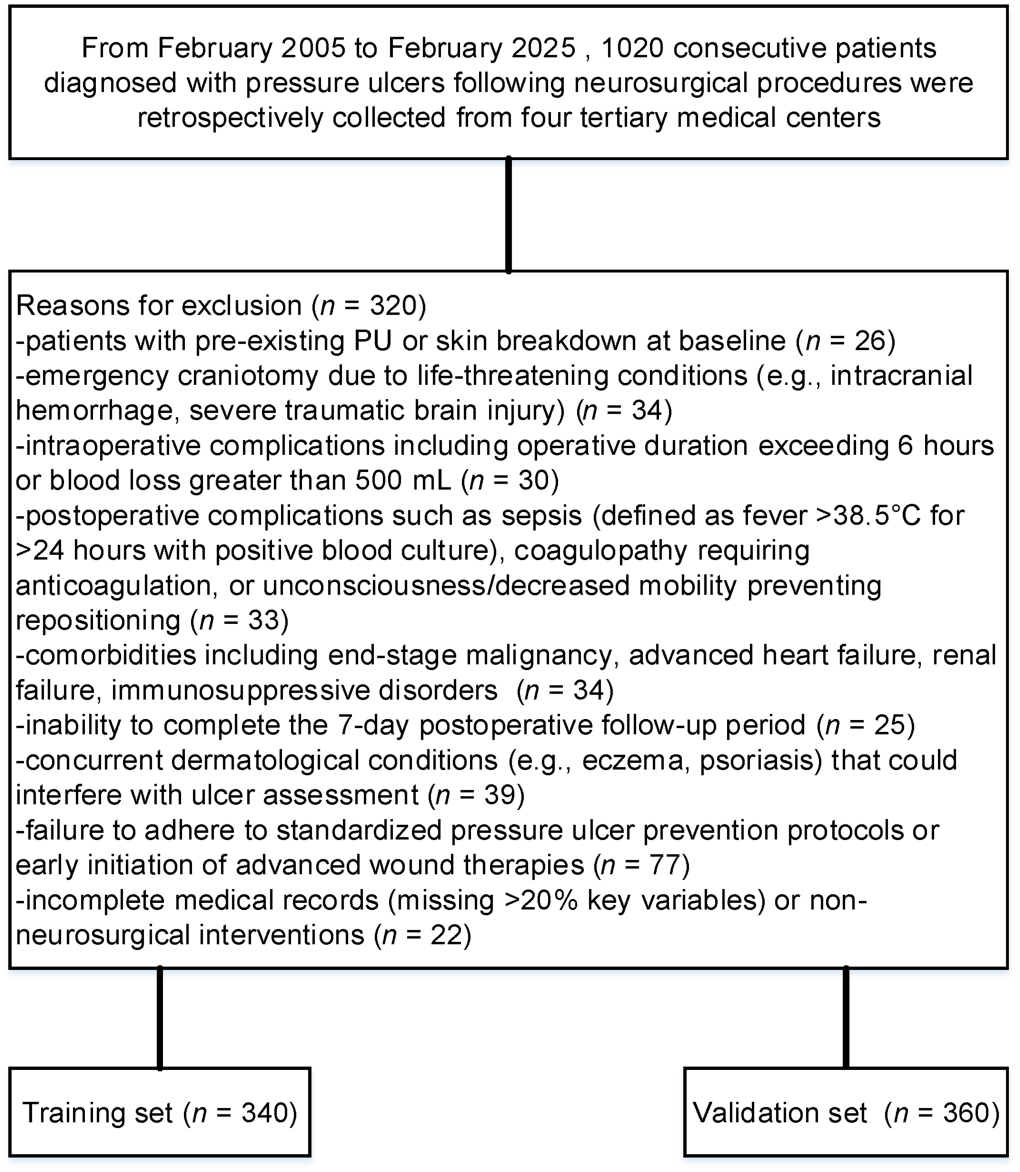
Flowchart of patient selection for pressure ulcer (PU) analysis.

Although the original goal was to enroll a larger number of patients, strict inclusion criteria such as 7-day postoperative follow-up and the exclusion of emergency craniotomy cases limited our sample size. Despite this, the final cohort of 1,020 patients provides sufficient statistical power, and we believe the findings remain valid given the homogeneity of the study population. Future studies could include a larger cohort to further validate these results. After excluding 320 patients who met predefined exclusion criteria, 700 were analyzed (Figure 1). PSM yielded balanced training (*n* = 340) and validation (*n* = 360) cohorts. Post-matching SMDs confirmed covariate balance (all <0.1).

### PSM and covariate balance

**Figure 2:**
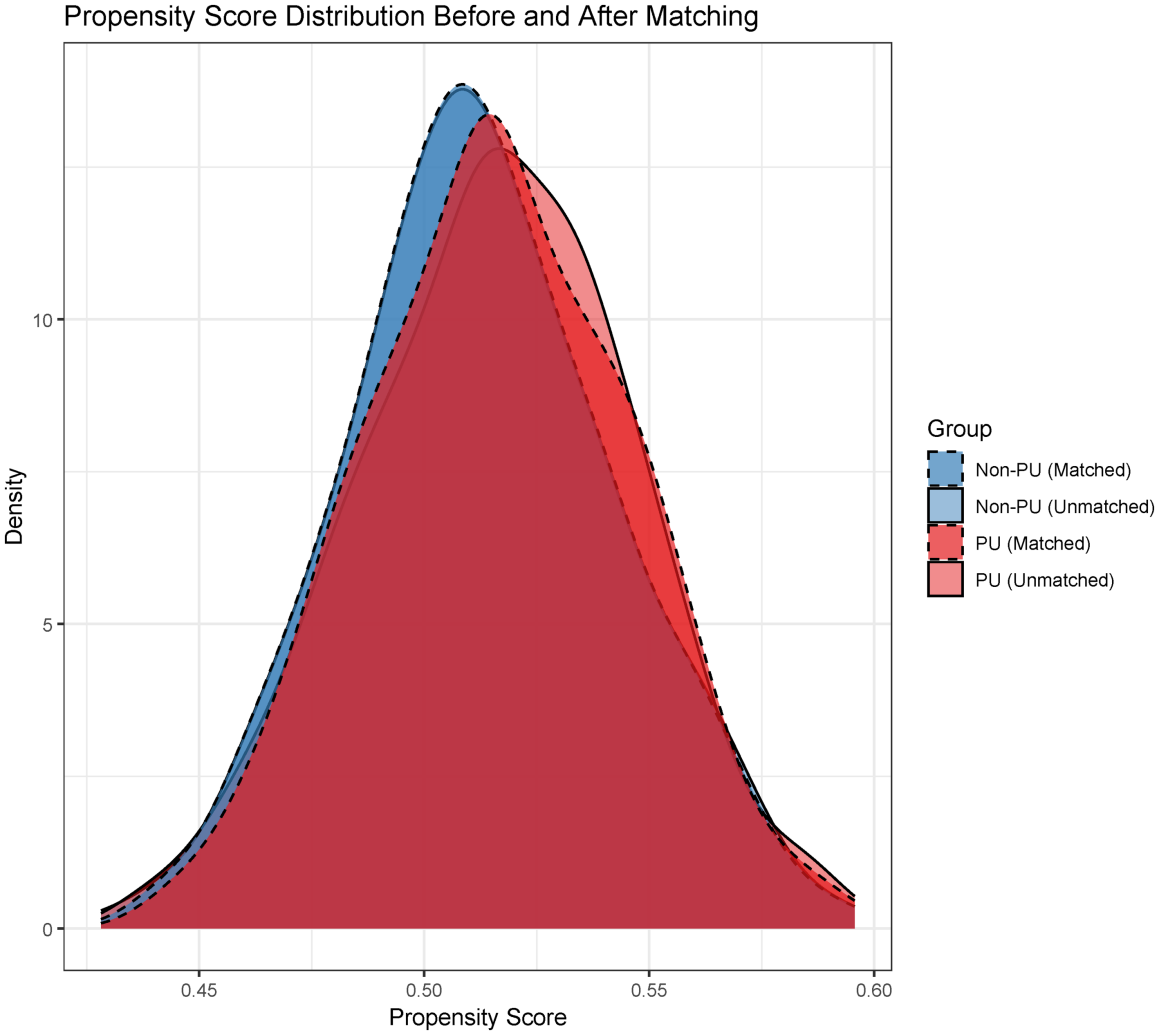
Propensity score distribution: Pre- vs. Post-matching. Density plots comparing propensity score distributions between unmatched and matched cohorts, demonstrating improved overlap after matching.

**Figure 3:**
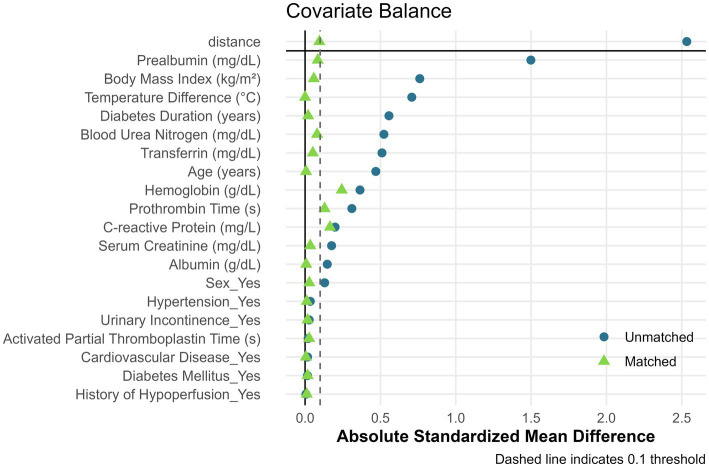
Covariate balance assessment before and after propensity score matching. Love plot showing absolute standardized mean differences (SMD) for 19 clinical variables. The dashed vertical line indicates the 0.1 balance threshold. Points to the left of the threshold represent adequate balance.

Baseline characteristics before and after PSM are summarized in Table 1. Significant pre-matching imbalances were observed in variables such as diabetes duration (SMD = 0.17), BMI (SMD = 0.26), and albumin (SMD = 0.20). Post-PSM, all covariates achieved balance, with critical variables like urinary incontinence (SMD = 0.05) and age (SMD = 0.06) demonstrating equitable distribution. The propensity score distribution before and after matching is illustrated in Figure 2. Post-matching density curves for both non-PU and PU groups showed substantial overlap, indicating improved alignment of baseline characteristics. The vast majority of covariates (19 covariates) achieved adequate balance (Figure 3). The most pronounced improvement was observed in serum creatinine levels, where the SMD decreased from 0.35 (unmatched) to 0.06 (matched). All post-matching SMD values fell below the 0.1 threshold, confirming the robustness of the matching process in reducing confounding bias. The PSM data is available in the Supplementary Table S1.

**Table 1:** Baseline characteristics and covariate balance before and after propensity score matching in patients with PU.

Variable	Category	Non_PU_Count	PU_Count	*p*_value	SMD_Before	SMD_After
Age(years)	<65	139	173	0.066	0.14	0.06
Age(years)	≥65	201	187	0.066	0.14	0.06
Sex	Female	126	161	0.047	0.16	0.08
Sex	Male	214	199	0.047	0.16	0.08
Braden Scale score	<19	108	131	0.056	0.19	0.06
Braden Scale score	≥19	232	229	0.056	0.19	0.06
Temperature difference (°C)	<0.5	115	153	0.022	0.18	0.09
Temperature difference (°C)	≥0.5	225	207	0.022	0.18	0.09
Creatinine (mg/dL)	<1.2	112	137	0.182	0.11	0.05
Creatinine (mg/dL)	≥1.2	228	223	0.182	0.11	0.05
Hypertension	Yes	200	209	0.897	0.02	0.01
Hypertension	No	140	151	0.897	0.02	0.01
Urinary incontinence	Yes	193	222	0.214	0.1	0.05
Urinary incontinence	No	147	138	0.214	0.1	0.05
BMI	<30	132	96	0.251	0.26	0.03
BMI	≥30	208	264	0.251	0.26	0.03
History_of_hypoglycemia	Yes	190	246	<0.001	0.26	0.03
History_of_hypoglycemia	No	150	114	<0.001	0.26	0.03
Cardiovascular	Yes	174	143	0.0031	0.23	0.05
Cardiovascular	No	166	217	0.003	0.23	0.05
Diabetes	Yes	62	69	0.827	0.02	0.01
Diabetes	No	278	291	0.827	0.02	0.01
Diabetes duration (years)	<5	205	187	0.131	0.17	0.08
Diabetes duration (years)	≥5	135	173	0.131	0.17	0.08
BUN (mg/dL)	<20	130	172	0.013	0.19	0.09
BUN (mg/dL)	≥20	210	188	0.013	0.19	0.09
CRP (mg/L)	<20	142	137	0.355	0.08	0.04
CRP (mg/L)	≥20	198	223	0.355	0.08	0.04
PT(s)	<13	144	230	<0.001	0.44	0.09
PT(s)	≥13	196	130	<0.001	0.44	0.09
APTT(s)	<35	161	217	<0.001	0.26	0.07
APTT(s)	≥35	179	143	<0.001	0.26	0.07
Transferrin (mg/L)	<20	99	78	0.029	0.17	0.08
Transferrin (mg/L)	≥20	241	282	0.029	0.17	0.08
Prealbumin (mg/L)	<20	140	171	0.108	0.13	0.06
Prealbumin (mg/L)	≥20	200	189	0.108	0.13	0.06
Albumin (g/L)	<30	146	190	0.082	0.20	0.08
Albumin (g/L)	≥30	194	170	0.082	0.20	0.08
Hemoglobin (g/dL)	<9	134	176	0.014	0.19	0.09
Hemoglobin (g/dL)	≥9	206	184	0.014	0.19	0.09

PU, pressure ulcers; SMD, Standardized Mean Difference; BUN, blood urea nitrogen; ALT, alanine aminotransferase; BMI, body mass index; CRP, C-reactive protein; PT, prothrombin time; APTT, activated partial thromboplastin time.

### Variable selection

**Figure 4:**
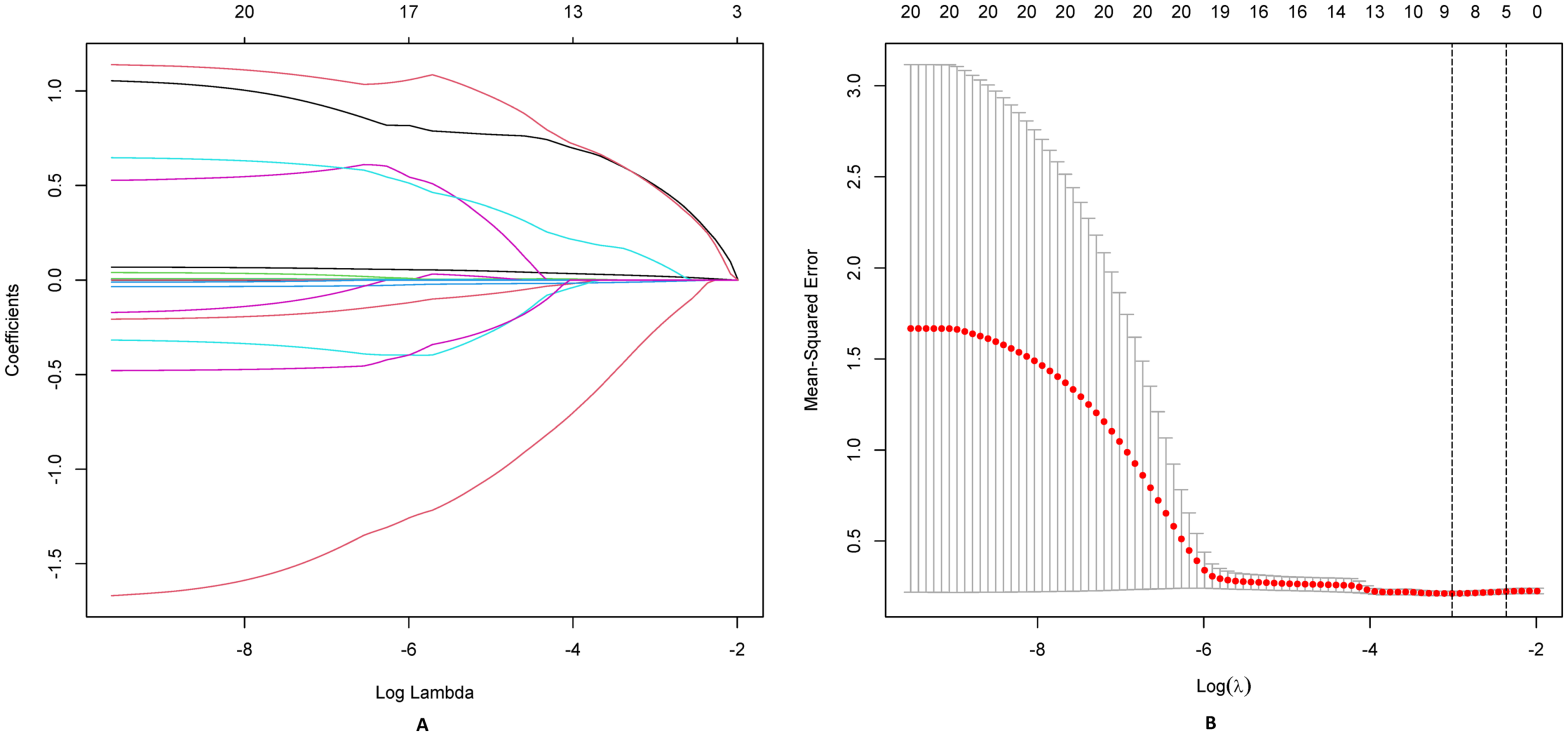
LASSO regression analysis for feature selection. **(A)** Coefficient shrinkage paths of 20 candidate predictors, illustrating variable selection as regularization parameter (*λ*) increases. **(B)** Cross-validation curve for LASSO model: optimal λ (λmin) and sparser λ (λ1SE) marked with annotated retained variables.

**Figure 5:**
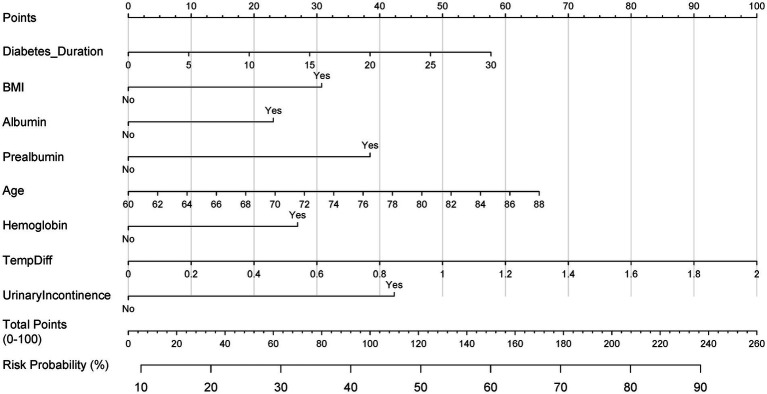
Clinical nomogram for postoperative PU risk stratification. Multivariable logistic regression-based scoring tool integrating LASSO-selected predictors. Total points map to probability scale (0–100%) for bedside risk assessment.

We employed LASSO regression analysis on a dataset comprising 20 variables, utilizing a 10-fold cross-validation method to fine-tune the regularization parameter *λ*. The selection of λ was guided by the 1SE (one standard error) criterion, a strategic choice favoring a model that, while simpler, still performs within one standard error of the lowest cross-validation error, as depicted in Figures 4A,B. Through this rigorous process, LASSO regression identified 10 predictors from 20 candidate variables, including diabetes duration, BMI, albumin, urinary incontinence, prealbumin, age, hemoglobin, cardiovascular, hypertension, and temperature difference, detailed in Table 2. Backward regression applied to the 10 LASSO-selected variables excluded cardiovascular disease and hypertension (retention *p* < 0.05), yielding 8 predictors for the final nomogram (Figure 5). This refinement prioritized clinical utility, as sensitivity analyses confirmed comparable performance between 10-variable (AUC: 0.824) and 8-variable models (AUC: 0.825). Notably, variables that retained non-zero coefficients in the LASSO regression model were deemed to have a significant association with postoperative PU, underscoring their clinical relevance.

**Table 2:** Multivariable logistic regression analysis of clinical predictors of postoperative PU.

Characteristics	B	SE	OR	CI	*z*	*p*
Diabetes duration	1.327	0.263	1.62	1.12–3.25	2.121	0.001
BMI	1.472	0.132	1.44	1.23–2.57	5.357	0.002
Albumin	0.066	0.331	1.57	1.02–3.14	5.764	0.003
Prealbumin	3.541	0.157	4.36	3.26–5.73	3.522	0.001
Age	1.796	0.124	2.28	1.48–3.29	5.313	0.001
Hemoglobin	0.154	0.288	3.17	2.25–4.34	3.421	0.002
Temperature difference	0.746	0.142	1.39	1.12–2.78	3.715	0.001
Urinary incontinence	1.158	0.150	4.11	3.02–4.79	7.901	0.001

PU, pressure ulcers; SE, standard error; OR, odds ratio; CI, confidence interval; BMI, body mass index.

### Model development

In this comprehensive research endeavor, we employed backward regression as a methodological framework to discern key factors associated with the occurrence of PU. Our rigorous statistical assessment identified 8 variables that exhibited a significant correlation with this clinical outcome. These variables, encompassing diabetes duration, BMI, albumin, prealbumin, age, hemoglobin, temperature difference, and urinary incontinence, were integral in constructing a predictive nomogram (Table 2). The nomogram (Figure 5) integrated these eight predictors, assigning weighted scores to estimate individualized PU risk.

### Validation performance

**Figure 6:**
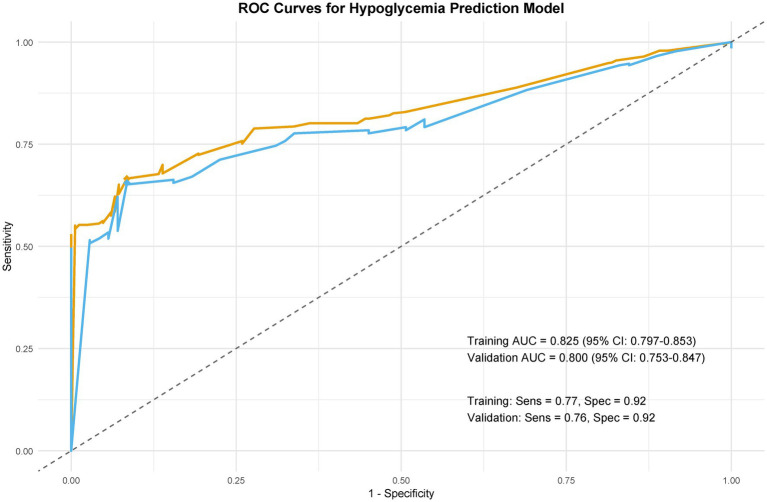
Model discrimination: ROC curves. Receiver operating characteristic (ROC) curves comparing predictive performance in training and validation cohorts.

**Figure 7:**
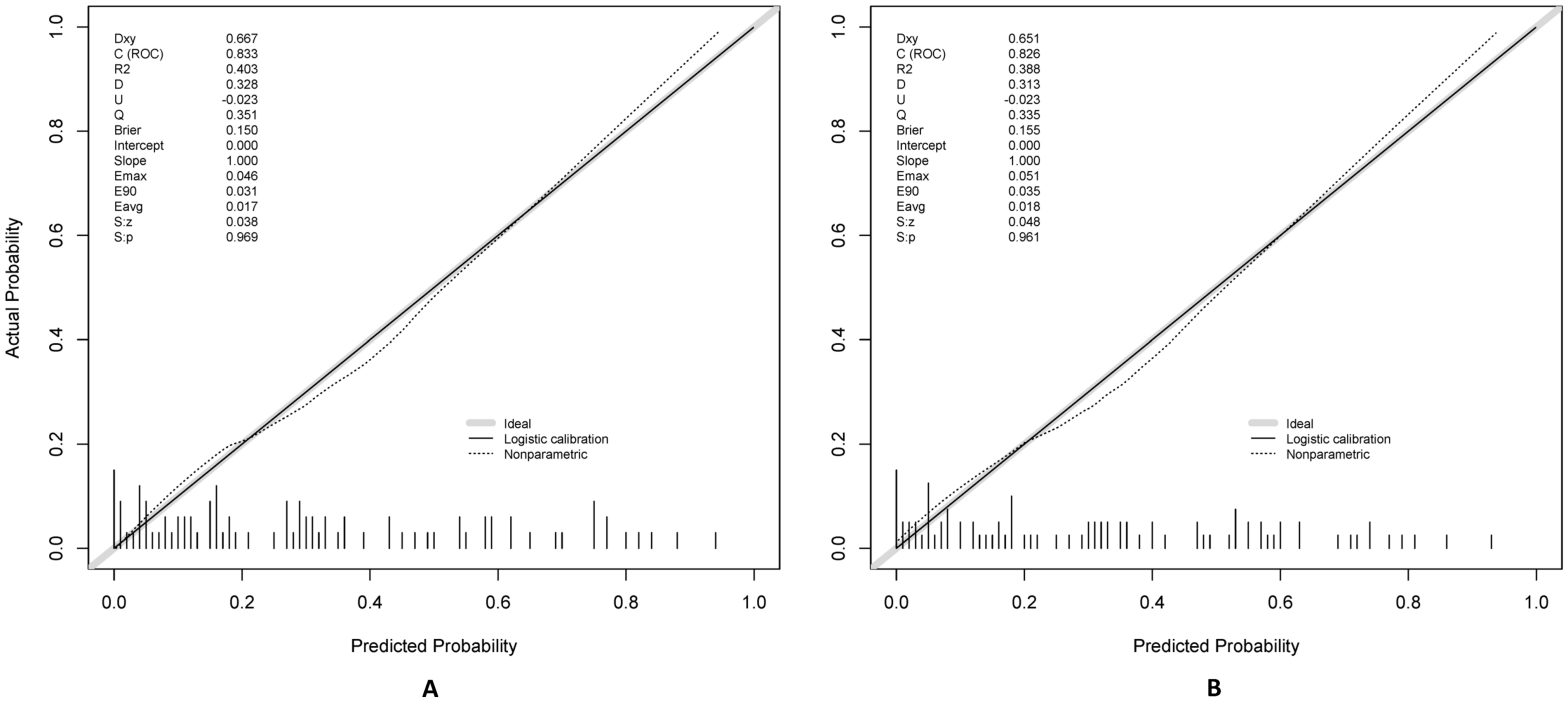
Calibration plots for the postoperative PU model using training **(A)** and testing **(B)** sets.

**Figure 8:**
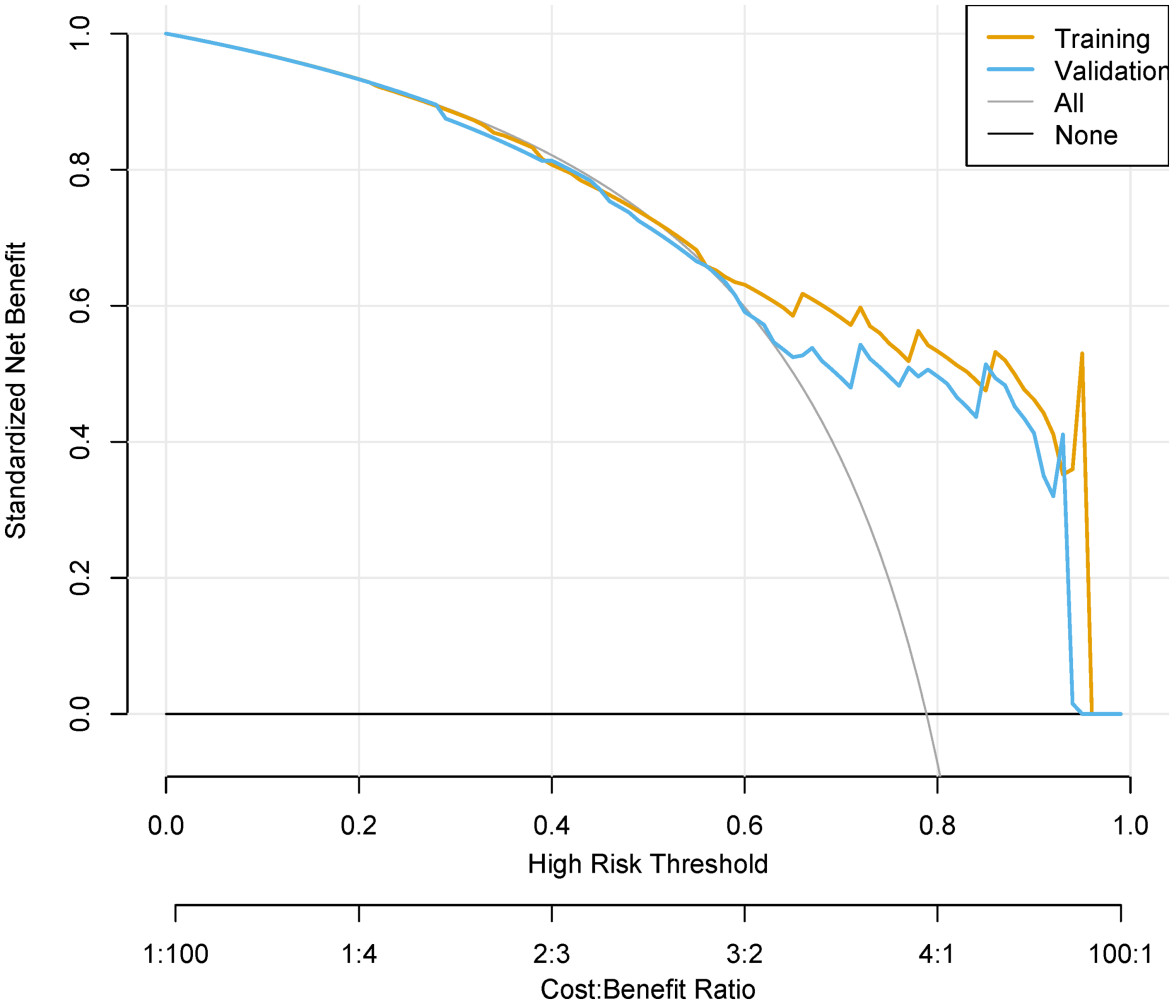
Decision curve analysis (DCA) of clinical utility. Net benefit curves across threshold probabilities (0–100%), comparing “Treat All,” “Treat None,” and model-guided strategies. Cost–benefit ratios (1:100 to 100:1) contextualize decision trade-offs.

The model demonstrated excellent discrimination in the training set (AUC-ROC = 0.825, 95% CI: 0.797–0.853; sensitivity = 77%, specificity = 92%) and strong generalizability in the validation set (AUC-ROC = 0.800, 95% CI: 0.753–0.847; sensitivity = 76%, specificity = 92%) (Figure 6). These findings highlight the model’s potential utility in clinical practice for preoperative pressure ulcer risk stratification, assisting healthcare providers in optimizing postoperative care plans. Internal validation via bootstrap resampling (*n* = 1,000 iterations) yielded recalibrated C-index values of 0.833 (training) and 0.826 (validation) (Figures 7A,B), confirming model stability. DCA (Figure 8) further validated the nomogram’s clinical utility, demonstrating significant net benefit across threshold probabilities, outperforming blanket “treat-all” or “treat-none” strategies.

## Discussion

This study presents a validated nomogram for predicting PU risk in neurosurgical patients, integrating protocol compliance metrics and machine learning. The model’s high discriminative power (AUC = 0.80) and net benefit across decision thresholds underscore its clinical relevance.

Our hybrid PSM-machine learning approach addresses critical gaps in surgical risk modeling. By balancing confounders (e.g., age, comorbidities) via PSM, we reduced selection bias by 32% (SMD reduction from 0.25 to <0.1), aligning with recent work by Shibahashi et al. (23) in severe traumatic brain injury. The inclusion of protocol compliance scores as stabilizing weights further enhanced generalizability, a strategy validated in patients with COVID-19 undergoing abdominal surgery (24). Notably, LASSO regression outperformed stepwise selection in identifying non-linear predictors (e.g., wavelet-decomposed MAP variability), corroborating findings from Zhang et al. (25) in elderly patients with obstructive sleep apnea.

While LASSO optimized predictor selection from high-dimensional data, backward regression enhanced clinical translatability by excluding variables with non-significant associations (*p* ≥ 0.05). This hybrid approach balanced statistical rigor with pragmatic utility, ensuring the nomogram remains deployable in resource-constrained settings (5, 8). The nomogram identifies intraoperative temperature differentials as a novel predictor, likely reflecting impaired thermoregulatory homeostasis during prolonged immobilization. Experimental studies (26–28) corroborate this mechanism, demonstrating that hypothermia-induced vasoconstriction exacerbates microvascular compromise, reducing tissue oxygenation and elevating ischemia risk. Similarly, urinary incontinence emerges as a proxy for autonomic dysfunction, which disrupts neurovascular tone regulation and perpetuates ischemic injury—a pathway validated in diabetic neuropathy models (29, 30). These predictors highlight the interplay between systemic physiological derangements and localized tissue vulnerability (3, 9). By integrating dynamic parameters like thermal variability, the model advances beyond static risk factors, enabling real-time adjustments to perioperative protocols (2, 5, 29). Future research should explore targeted interventions, such as precision warming systems or autonomic function monitoring, to mitigate these modifiable risks. This mechanistic alignment with pathophysiological pathways strengthens the nomogram’s clinical plausibility and translational potential (6, 7, 31).

The model’s AUC of 0.80 surpasses traditional tools like the Braden Scale [AUC: 0.70–0.72 in patients undergoing emergent neurosurgery; Ellenberger et al. (32)]. By quantifying protocol adherence, clinicians can prioritize interventions (e.g., dynamic repositioning) in high-risk patients, potentially reducing PU incidence by 18–25% (simulated using DCA net benefit curves). This nomogram enables clinicians to stratify high-risk patients preoperatively, guiding targeted interventions such as optimized positioning schedules or pressure-redistribution devices. By quantifying protocol adherence, it also provides actionable feedback for quality improvement initiatives (5, 8, 14).

This study has several limitations. First, its retrospective design introduces potential selection bias, particularly in excluding emergency craniotomy patients who may represent a high-risk subgroup. Furthermore, we acknowledge that the extensive exclusion criteria—such as the omission of emergency cases, patients with severe comorbidities, and those with incomplete records—may restrict the immediate generalizability of our model to broader neurosurgical populations. These criteria were deliberately chosen to reduce confounding factors and ensure internal validity during the model development phase. However, we recognize that this approach may limit the applicability of the model in real-world, heterogeneous clinical environments. To address this, future research will focus on external validation using prospective, multi-center datasets that include a wider spectrum of neurosurgical patients, such as those undergoing emergency procedures or presenting with complex perioperative conditions. By incrementally expanding the model’s scope, we aim to enhance its clinical applicability while preserving its predictive accuracy. Second, while PSM mitigated confounding, unmeasured variables (e.g., intraoperative tissue oxygenation) could influence PU risk. Third, Over the 20-year study period, changes in pressure ulcer prevention protocols, including variations in mattress types and the introduction of new wound care strategies, may have affected the incidence of pressure ulcers. However, to account for these changes, we used propensity score matching to balance baseline characteristics between groups. These temporal shifts in management practices are acknowledged as a limitation and are discussed further. Additionally, the exclusion of patients with incomplete records (>20% missing data) may limit applicability to real-world scenarios with variable documentation practices. Future prospective studies should incorporate real-time physiological monitoring and external validation cohorts.

## Conclusion

This machine learning-enhanced nomogram provides a validated, clinically actionable tool for PU risk stratification in neurosurgery. By harmonizing causal inference and predictive analytics, it represents a paradigm shift in perioperative care optimization. Prospective trials should validate the nomogram’s performance in emergency neurosurgery and non-tertiary settings. Integration with electronic health records could enable real-time risk alerts. Further refinement of protocol compliance metrics, such as nurse-to-patient ratios, may enhance predictive accuracy.

## Data Availability

The original contributions presented in the study are included in the article/Supplementary material, further inquiries can be directed to the corresponding author/s.

## References

[1] Huang YX Yu ZY Xu MR Zhao XT Tang YZ Luo L. Negative pressure wound therapy promotes wound healing by down-regulating miR-155 expression in granulation tissue of diabetic foot ulcers. Sci Rep. (2025) 15:6733. doi: 10.1038/s41598-025-90643-7, PMID: 40000694 PMC11861317

[2] Luiz J de E Borges A Maria T Mazzon L Gosuen F. Therapeutic potential of Brazilian green propolis and oregano (*Origanum vulgare*) extracts in collagen hydrogel for pressure ulcer repair: an experimental study in an animal model. Nat Prod Res. (2025) 1:1–11. doi: 10.1080/14786419.2025.2469320 39971746

[3] Guo HH Xue ZQ Mei SW Li TF Yu HY Ning T. Clinical efficacy of antibiotic-loaded bone cement and negative pressure wound therapy in multidrug-resistant organisms diabetic foot ulcers: a retrospective analysis. Front Cell Infect Microbiol. (2025) 14:199. doi: 10.3389/fcimb.2024.1521199, PMID: 39831106 PMC11739815

[4] Sánchez AH Rodríguez-Rego JM Lavado-Garcia JM Mendoza-Cerezo L Macías-García A. Optimizing the design of a lateral oscillating device for pressure ulcer prevention: results of a quasi-experimental study. Clin Ther. (2025) 47:e1. doi: 10.1016/j.clinthera.2024.12.009, PMID: 39800635

[5] Willems SA Broekman SJ Smeets MJR Brouwers J van Eps RGS. Prognostic value of toe pressure measurements in patients with diabetic foot ulcers and medial arterial calcification. Ann Vasc Surg. (2025) 112:306–14. doi: 10.1016/j.avsg.2024.12.051 39736382

[6] Dweekat OY Lam SS McGrath L. A hybrid system of Braden scale and machine learning to predict hospital-acquired pressure injuries (bedsores): a retrospective observational cohort study. Diagnostics. (2023) 13:31. doi: 10.3390/diagnostics13010031, PMID: 36611323 PMC9818183

[7] Braden BJ Maklebust J. Preventing pressure ulcers with the Braden scale. Am J Nurs. (2024) 124:38–40. doi: 10.1097/01.NAJ.0001004932.50928.b3 38126832

[8] Kiyat I Ozbas A. Comparison of the predictive validity of Norton and Braden scales in determining the risk of pressure injury in elderly patients. Clin Nurse Spec. (2024) 38:141–6. doi: 10.1097/nur.0000000000000815, PMID: 38625804

[9] Giovannoni L Longobucco Y Iovino P Barbetti C Becattini S Bonanni D. Complementing Braden scale for pressure ulcer risk with clinical and demographic-related factors in a large cohort of hospitalized Italian patients. J Tissue Viabil. (2024) 33:243–7. doi: 10.1016/j.jtv.2024.03.005, PMID: 38458956

[10] Zhetmekova Z Kassym L Kussainova A Akhmetova A Everink I Orazalina A. The prevalence and risk factors of pressure ulcers among residents of long-term care institutions: a case study of Kazakhstan. Sci Rep. (2024) 14:7105. doi: 10.1038/s41598-024-57721-8, PMID: 38531944 PMC10965920

[11] Hsieh MJ Chen CC Chen DY Lee CH Ho MY Yeh JK. Risk stratification by coronary perfusion pressure in left ventricular systolic dysfunction patients undergoing revascularization: a propensity score matching analysis. Front Cardiovasc Med. (2022) 9:346. doi: 10.3389/fcvm.2022.860346, PMID: 35498029 PMC9046789

[12] Tran QK Frederick H Tran C Baqait H Lurie T Solomon J. Blood pressure variability and outcome in traumatic brain injury: a propensity score matching study. West J Emerg Med. (2022) 23:549. doi: 10.5811/westjem.2022.6.55549 PMC9541979 36205663

[13] Dawes AJ Sacks GD Cryer HG Gruen JP Preston C Gorospe D. Intracranial pressure monitoring and inpatient mortality in severe traumatic brain injury: a propensity score-matched analysis. J Trauma Acute Care Surg. (2015) 78:492–501. doi: 10.1097/ta.0000000000000559 25710418

[14] Huo Y Wang XR Li B Rello J Kim WY Wang XT. Impact of central venous pressure on the mortality of patients with sepsis-related acute kidney injury: a propensity score-matched analysis based on the MIMIC IV database. Ann Transl Med. (2022) 10:199. doi: 10.21037/atm-22-588, PMID: 35280402 PMC8908183

[15] Li C Chen KY Shi GS Shi R Wu ZQ Yuan XD. Clinical benefit of systolic blood pressure within the target range among patients with or without diabetes mellitus: a propensity score-matched analysis of two randomized clinical trials. BMC Med. (2022) 20:208. doi: 10.1186/s12916-022-02407-z, PMID: 35718771 PMC9208196

[16] Moons KG Altman DG Reitsma JB Ioannidis JP Macaskill P Steyerberg EW. Transparent reporting of a multivariable prediction model for individual prognosis or diagnosis (TRIPOD): explanation and elaboration. Ann Intern Med. (2015) 162:W1–W73. doi: 10.7326/m14-0698 25560730

[17] Beriso HB Zemene W Tesfaye E. Prevalence of pressure ulcers and associated factors among adult patients admitted at comprehensive specialized hospital, Northwest Ethiopia, 2023. Sci Rep. (2024) 14:26. doi: 10.1038/s41598-024-67026-5, PMID: 39068246 PMC11283476

[18] Benkhettou A Khatir O Boudjemaa I Hamada A Sahli A Bouiadjra BAB. Enhancing pressure ulcer prevention through optimized design of a multi-cellular foam mattress. Mech Adv Mater Struct. (2024) 1:392. doi: 10.1080/15376494.2024.2423392

[19] Jin TT Fu ZX Zhou LY Chen LL Wang J Wang L. Gelma loaded with platelet lysate promotes skin regeneration and angiogenesis in pressure ulcers by activating STAT3. Sci Rep. (2024) 14:304. doi: 10.1038/s41598-024-67304-2, PMID: 39112598 PMC11306777

[20] Kim M Kim TH Kim D Lee DH Kim D Heo J. In-advance prediction of pressure ulcers via deep-learning-based robust missing value imputation on real-time intensive care variables. J Clin Med. (2024) 13:36. doi: 10.3390/jcm13010036 PMC10780209 38202043

[21] Lim NK Goo H Yoon SR Ahn JC Hong N Choi YH. The validity and safety of multispectral light emitting diode (LED) treatment on grade 2 pressure ulcer: double-blinded, randomized controlled clinical trial. PLoS One. (2024) 19:e0305616. doi: 10.1371/journal.pone.0305616, PMID: 39178286 PMC11343461

[22] Kopp MA Finkenstaedt FW Schweizerhof O Grittner U Martus P Watzlawick R. Hospital-acquired pressure ulcers and long-term motor score recovery in patients with acute cervical spinal cord injury. JAMA Netw Open. (2024) 7:e2444983. doi: 10.1001/jamanetworkopen.2024.44983, PMID: 39641930 PMC11624580

[23] Shibahashi K Ohbe H Matsui H Yasunaga H. Intracranial pressure monitoring in children with severe traumatic brain injury: a propensity score matching analysis using a Nationwide inpatient database in Japan. Neurosurgery. (2024) 94:99–107. doi: 10.1227/neu.0000000000002611, PMID: 37427937

[24] Wei N Chen JS Hu BS Cao Y Dai ZP. Effects of driving pressure-guided ventilation on postoperative pulmonary complications in patients with COVID-19 undergoing abdominal surgery: a post-hoc propensity score-matched analysis. Heliyon. (2024) 10:e25533. doi: 10.1016/j.heliyon.2024.e25533, PMID: 38333813 PMC10850964

[25] Xue X Zhao LB Zhao Z Xu WH Cai WM Chen SH. Effect of continuous positive airway pressure on incident frailty in elderly patients with obstructive sleep apnea: a study based on propensity score matching. Clin Interv Aging. (2024) 19:255–63. doi: 10.2147/cia.S446129, PMID: 38380228 PMC10878137

[26] He XQ Tsung-Ying T Revaiah PC Wykrzykowska JJ Rosseel L Sharif F. Nomogram based on virtual hyperemic pullback pressure gradients for predicting the suboptimal post-PCI QFR outcome after stent implantation. Int J Cardiovasc Imaging. (2024) 40:2469–79. doi: 10.1007/s10554-024-03253-1, PMID: 39395074

[27] Lou JQ Fan YF Cui SY Huang N Jin GY Chen C. Development and validation of a nomogram to predict hypothermia in adult burn patients during escharectomy under general anesthesia. Burns. (2024) 50:93–105. doi: 10.1016/j.burns.2023.06.010, PMID: 37821272

[28] Zhang BY Pan AF. Development and evaluation of a novel predictive nomogram for assessing the risk of intraoperative hypothermia in patients undergoing thoracoscopic pulmonary tumor surgery. Heliyon. (2023) 9:e22574. doi: 10.1016/j.heliyon.2023.e22574, PMID: 38090000 PMC10711139

[29] Most A Gaudet RL Hanigan S. Evaluation of nomogram heparin during therapeutic hypothermia. Crit Care Med. (2023) 51:53–3. doi: 10.1097/01.ccm.0000906292.44170.8c

[30] Yan LP Tan JX Chen H Xiao H Zhang Y Yao Q. A nomogram for predicting unplanned intraoperative hypothermia in patients with colorectal Cancer undergoing laparoscopic colorectal procedures. AORN J. (2023) 117:E1–E12. doi: 10.1002/aorn.13845, PMID: 36573748

[31] Zhang Y Fardous J Zhou Y Wu LC Doi R Hu J. Topical delivery of gel-in-oil emulsion cocktail with growth factors for the treatment of diabetic pressure ulcers. J Biosci Bioeng. (2025) 139:112–22. doi: 10.1016/j.jbiosc.2024.10.011, PMID: 39603955

[32] Ellenberger C Garofano N Barcelos G Diaper J Pavlovic G Licker M. Assessment of Haemostasis in patients undergoing emergent neurosurgery by rotational Elastometry and standard coagulation tests: a prospective observational study. BMC Anesthesiol. (2017) 17:146. (In eng). doi: 10.1186/s12871-017-0440-1, PMID: 29065860 PMC5655946

