# Supplementary material for: Development and validation of a perioperative risk prediction model for pressure ulcers in neurosurgical procedures: a machine learning approach with protocol compliance metrics
Source: Front Med (Lausanne). 2025 Jul 2;12:1600481. doi: 10.3389/fmed.2025.1600481 (PMC12263567; doi:10.3389/fmed.2025.1600481)
Supplement: Supplementary file 1 [file Table_1.DOCX]

| variable | level | PU | Non-PU | SMD |
| --- | --- | --- | --- | --- |
|  |  | 360 | 340 |  |
| ASD |  | 5561871 | 5615783 | 0064 |
| BUNSD |  | 1974284 | 1929278 | 0159 |
| TIQR |  | 030[030030 | 030[030030 | 0061 |
| CSD |  | 091014 | 091015 | 0016 |
| BMISD |  | 2268323 | 2260332 | 0026 |
| CRPSD |  | 450067 | 445065 | 0074 |
| PTSD |  | 1220171 | 1244171 | 0140 |
| APTTSD |  | 2879431 | 2851347 | 0073 |
| TSD |  | 237923937 | 241504092 | 0089 |
| ASD |  | 376050 | 377060 | 0024 |
| HSD |  | 1358168 | 1301214 | 0296 |
| PSD |  | 2481292 | 2413339 | 0215 |
| DSD |  | 473068 | 473075 | 0013 |
| SD |  | 042026 | 051027 | 0363 |
| SD |  | 100035 | 100000 | <0001 |
| Sx | N | 78605 | 52584 | 0042 |
| X | Y | 51395 | 37416 |  |
| H | N | 65504 | 46517 | 0026 |
| X1 | Y | 64496 | 43483 |  |
| U | N | 108837 | 75843 | 0015 |
| X2 | Y | 21163 | 14157 |  |
| H | N | 85659 | 58652 | 0015 |
| X3 | Y | 44341 | 31348 |  |
| C | N | 115891 | 79888 | 0012 |
| X4 | Y | 14109 | 10112 |  |
| D | N | 115891 | 81910 | 0062 |
| X5 | Y | 14109 | 890 |  |
|  | 1 | 216 | 111 | 0347 |
| X6 | 2 | 216 | 111 |  |
| X7 | 3 | 108 | 111 |  |
| X8 | 4 | 216 | 111 |  |
| X9 | 5 | 216 | 111 |  |
| X10 | 6 | 108 | 111 |  |
| X11 | 7 | 216 | 111 |  |
| X12 | 8 | 216 | 111 |  |
| X13 | 9 | 108 | 111 |  |
| X14 | 10 | 216 | 111 |  |
| X15 | 11 | 216 | 111 |  |
| X16 | 12 | 216 | 111 |  |
| X17 | 13 | 108 | 111 |  |
| X18 | 14 | 108 | 111 |  |
| X19 | 15 | 108 | 111 |  |
| X20 | 16 | 216 | 111 |  |
| X21 | 17 | 216 | 111 |  |
| X22 | 18 | 216 | 111 |  |
| X23 | 19 | 216 | 111 |  |
| X24 | 20 | 216 | 111 |  |
| X25 | 21 | 108 | 111 |  |
| X26 | 22 | 216 | 111 |  |
| X27 | 23 | 108 | 111 |  |
| X28 | 24 | 216 | 111 |  |
| X29 | 25 | 216 | 111 |  |
| X30 | 26 | 108 | 111 |  |
| X31 | 27 | 108 | 111 |  |
| X32 | 28 | 108 | 111 |  |
| X33 | 29 | 108 | 111 |  |
| X34 | 30 | 216 | 111 |  |
| X35 | 31 | 108 | 111 |  |
| X36 | 32 | 216 | 111 |  |
| X37 | 33 | 216 | 111 |  |
| X38 | 34 | 216 | 111 |  |
| X39 | 35 | 216 | 111 |  |
| X40 | 36 | 108 | 111 |  |
| X41 | 37 | 216 | 111 |  |
| X42 | 38 | 108 | 111 |  |
| X43 | 39 | 108 | 111 |  |
| X44 | 40 | 108 | 111 |  |
| X45 | 41 | 108 | 111 |  |
| X46 | 42 | 108 | 111 |  |
| X47 | 43 | 108 | 111 |  |
| X48 | 44 | 216 | 111 |  |
| X49 | 45 | 108 | 111 |  |
| X50 | 46 | 108 | 111 |  |
| X51 | 47 | 216 | 111 |  |
| X52 | 48 | 216 | 111 |  |
| X53 | 49 | 216 | 111 |  |
| X54 | 50 | 108 | 111 |  |
| X55 | 51 | 216 | 111 |  |
| X56 | 52 | 216 | 111 |  |
| X57 | 53 | 216 | 111 |  |
| X58 | 54 | 108 | 111 |  |
| X59 | 55 | 108 | 111 |  |
| X60 | 56 | 108 | 111 |  |
| X61 | 57 | 216 | 111 |  |
| X62 | 58 | 216 | 111 |  |
| X63 | 59 | 216 | 111 |  |
| X64 | 60 | 108 | 111 |  |
| X65 | 61 | 108 | 111 |  |
| X66 | 62 | 216 | 111 |  |
| X67 | 63 | 216 | 111 |  |
| X68 | 64 | 108 | 111 |  |
| X69 | 65 | 216 | 111 |  |
| X70 | 66 | 108 | 111 |  |
| X71 | 67 | 108 | 111 |  |
| X72 | 68 | 108 | 111 |  |
| X73 | 69 | 216 | 111 |  |
| X74 | 70 | 108 | 111 |  |
| X75 | 71 | 108 | 111 |  |
| X76 | 72 | 108 | 111 |  |
| X77 | 73 | 108 | 111 |  |
| X78 | 74 | 216 | 111 |  |
| X79 | 75 | 108 | 111 |  |
| X80 | 76 | 108 | 111 |  |
| X81 | 77 | 108 | 111 |  |
| X82 | 78 | 108 | 111 |  |
| X83 | 79 | 108 | 111 |  |
| X84 | 80 | 108 | 111 |  |
| X85 | 81 | 108 | 111 |  |
| X86 | 82 | 216 | 111 |  |
| X87 | 83 | 108 | 111 |  |
| X88 | 84 | 108 | 111 |  |
| X89 | 85 | 108 | 111 |  |
| X90 | 86 | 108 | 111 |  |
| X91 | 87 | 216 | 111 |  |
| X92 | 88 | 108 | 111 |  |
| X93 | 89 | 108 | 111 |  |
